# Association of Meat Subtypes With Colorectal Polyp Prevalence: Finding From the Lanxi Pre-colorectal Cancer Cohort in China

**DOI:** 10.3389/fnut.2022.833571

**Published:** 2022-03-18

**Authors:** Xiaoyin Chai, Yin Li, Zihan Yin, Fei Wu, Peiling Hu, Xiaohui Liu, Shuhan Tong, Pan Zhuang, Yu Zhang, Weifang Zheng, Jingjing Jiao

**Affiliations:** ^1^Lanxi Red Cross Hospital, Jinhua, China; ^2^Department of Nutrition, School of Public Health, Zhejiang University School of Medicine, Hangzhou, China; ^3^Zhejiang Key Laboratory for Agro-Food Processing, Department of Food Science and Nutrition, College of Biosystems Engineering and Food Science, Fuli Institute of Food Science, Zhejiang University, Hangzhou, China; ^4^Lanxi Hospital of Traditional Chinese Medicine, Jinhua, China

**Keywords:** colorectal polyps, meat consumption, poultry consumption, meat subtypes, Lanxi Pre-colorectal Cancer Cohort

## Abstract

**Background:**

Although the detrimental effect of red meat on colorectal cancer (CRC) incidence has been extensively reported, no previous studies have comprehensively linked different meat subtypes with colorectal polyp occurrence. The aim was to assess the association of meat and subtypes with colorectal polyp prevalence for the high-risk CRC Chinese population. Besides, we also focused on the association according to sizes, subsites, and multiplicity of polyps.

**Methods:**

High-risk CRC patients aged 40–80 years were enrolled into the Lanxi Pre-colorectal Cancer Cohort (LP3C) between March 2018 and December 2019. Cross-sectional analyses were conducted by using the baseline data from LP3C. A validated food frequency questionnaire (FFQ) was employed to collect dietary information. Odds ratios (ORs) and 95% confidence intervals (95% CIs) of colorectal polyp prevalence were estimated by multivariate logistic regression.

**Results:**

2,064 colorectal polyp cases were identified among 6,783 eligible participants in the survey of LP3C (March 2018 and December 2019). Total meat intake was positively related to rectum polyp prevalence (*P*_for trend_ = 0.01) but was not linked to total colorectal polyps after multivariable adjustment. For meat subtypes, higher poultry consumption was significantly related to a higher polyp prevalence [OR_Q4vs.Q1_ (95% CI): 1.20 (1.02–1.42); *P*_for trend_ = 0.03]. Processed red meat intake was linked to an increased small polyp prevalence (*P*_for trend_ = 0.03) while unprocessed red meat had a relation with a higher rectum polyp prevalence (*P*_for trend_ = 0.04). Furthermore, seafood intake had a significant association with a higher multiple polyp prevalence [OR_Q4vs.Q1_ (95% CI): 1.70 (1.31–2.21); *P*_for trend_ < 0.001].

**Conclusion:**

The finding was that poultry meat consumption was related to a higher polyp prevalence. Besides, total meat consumption, processed and unprocessed red meat consumption, seafood consumption had a positive relation with certain polyp subtypes prevalence. Generally recommending reducing total meat consumption, including poultry, processed and unprocessed red meat, and seafood intake, may prevent colorectal polyps.

## Introduction

It was announced that one of the top five causes of cancer-related death was colorectal cancer (CRC) in China. Meanwhile, China is undergoing a cancer transition period with an increasing burden of CRC (1). Increased incidence of CRC may be due to adaptation to a Western lifestyle after rapid economic transformations, such as lower fiber intake and higher red meat intake (2). Previous studies revealed a significant relation of red meat consumption with colon cancer risk in the Nurses’ Health Study and Health Professionals Follow-Up Study (3, 4).

Colorectal adenomas and polyps that were regarded as precursor lesions of CRC, attracted more and more attention. More and more studies shed light on the risk factors of adenomas and polyps. Higher total meat consumption and higher red meat consumption were both positively related to an increased hyperplastic polyp risk (5). Furthermore, a meta-analysis involving twenty-seven studies found that individuals with higher meat consumption or higher processed meat consumption had increased colorectal adenoma risk. Besides, the dose-response relationship was also found for meat consumption (per 100 g/day) in the meta-analysis (6). A non-linear relation for red meat consumption was shown in another meta-analysis (7). However, red meat consumption was not related to adenoma prevalence according to a large screening study (8).

Among all subtypes of meat, the relation of processed red meat or total red meat with polyp risk was widely examined, whereas few previous population-based studies have comprehensively explored the relation of poultry intake or fish consumption with polyp risk. No relation of poultry meat consumption and fish consumption with colorectal adenoma risk was found in a meta-analysis (9), while a positive association was observed for higher intake of poultry among Japanese-Brazilian population (10). There were inconsistent findings on the relation of poultry meat intake and fish consumption with colorectal cancer risk. A meta-analysis revealed a weak protection role for fish consumption but no association for poultry intake (11). However, it was not found a significant relation in the UK Biobank study for both fish and poultry intake (12).

Importantly, previous studies were largely conducted in Western populations whereas little was known among Asian populations, such as Chinese undergoing “Westernization” of diets. Therefore, evidence is needed for Chinese individuals about the role of meat and meat subtypes consumption on colorectal polyp risk. With the aim of filling the gap in the knowledge, we employed data from the Lanxi Pre-colorectal Cancer Cohort (LP3C) to investigate the relation of meat consumption with colorectal polyp prevalence.

## Materials and Methods

### Study Design and Study Population

The LP3C study began in March 2018 in Lanxi, Jinhua, Zhejiang Province, China. High-risk CRC patients aged 40–80 years were enrolled from the Lanxi of 16 government-administered units. Participants were required to complete assessment questionnaire of the CRC risk factor, which included a personal history of colorectal polyps or CRC, family history of CRC, and a fecal occult blood test. Each participant was required to complete two fecal occult blood tests with an interval of a week. The participants who had family or personal history and/or received a positive outcome from the above fecal test were considered as individuals at a high risk of CRC (13). They were required to participate colonoscopy at Lanxi Red Cross Hospital within a month. All informed consent participants needed to complete a questionnaire by the interviewer face-to-face before the colonoscopy exam, including general socio-economic information, demographic, diet, and lifestyle characteristics. The study design and methodology have been described in a published article (14).

**FIGURE 1:**
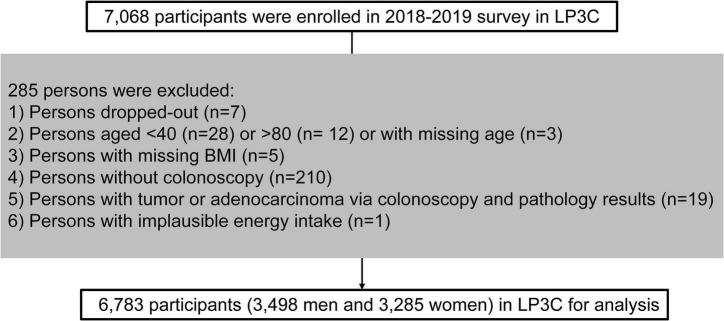
Flow chart of study participants in the current LP3C study.

A cross-sectional analysis was performed by utilizing the baseline data in the LP3C study (March 2018 and December 2019). The survey enrolled 7,068 participants in total. Exclusion criteria: withdrew (*n* = 7), aged < 40 or > 80 (*n* = 40), missing age (*n* = 3), missing BMI (*n* = 5) and missing colonoscopy results (*n* = 210), adenocarcinoma or other malignant tumors patients (*n* = 19), and implausible energy intake (*n* = 1). Finally, 6,783 eligible participants were included in the cross-sectional analysis (3,498 men and 3,285 women) (Figure 1).

### Dietary Assessment and Covariates

A food frequency questionnaire (FFQ) of China Kadoorie Biobank was modified by adding three food groups (spicy food, processed and unprocessed red meat), and then we used the modified FFQ to achieve dietary data (15). The 15 food groups included staple food (rice, wheat, other), meat (fish or seafood, poultry, processed red meat, and unprocessed red meat), soybean products, fresh vegetables, preserved vegetables, fresh fruits, dairy products (milk or fermented), eggs and spicy food; each with 5 frequency level of consumption (never, yearly, monthly, weekly, and daily). The FFQ proved validated by evaluating the correlation of food intake measured by FFQ with two 24-h dietary recalls. A strong correlation of total energy intake by two measurement methods was shown in the published article (14). Estimating food portion size was through food model atlas and measuring glasses, jars, and bowels. Total energy consumption was computed via the China Food Composition Tables (2018) (16). The overall diet quality of participants was evaluated by a healthy diet score (17). Overall 10 key dietary components were considered in the healthy diet score, including whole grain consumption (≥ median), preserved vegetables consumption (< median), vegetables consumption (≥ median), fruit consumption (≥ median), unprocessed red meat consumption (<median), fish or seafood consumption (≥ median), processed red meat consumption (< median), dairy products consumption (≥ median), refined grain consumption (< median) and tea consumption (≥ median). Participants would get one point for each qualified intake of dietary components and the sum of all these components score was defined as the total healthy diet score ranging from 0 to 10. When we focused on the relation of certain food with the prevalence of colorectal polyps, the healthy diet score minus the score of the dietary components was used in the current analysis.

Detailed information (e.g., demographic characteristics, lifestyle factors, personal and family medical history, and drug use) were also obtained through self-reporting. Anthropometric methods were used to measure weight and height. The Compendium of Physical Activities aided us to calculate metabolic equivalent task hours per week (MET-h/wk) of physical activity (18). Body weight (kg) divided by height (m) squared was defined as body mass index (BMI). A person’s lifestyle quality was represented by a healthy lifestyle score (19). The healthy lifestyle score was comprised of a BMI ≥ 18.5 and ≤ 24 kg/m^2^, never smoking, a healthy diet score >40th percentile, physical activity for ≥ 30 min/d of moderate-to-vigorous intensity activity, and alcohol intake (> 0 and < 14 g/d for females, < 28 g/d for males). The sum of the five components score was made of a healthy lifestyle. If any one of the above scoring criteria was qualified, participants would be given one point. The range of healthy lifestyle score was 0–5.

### Ascertainment of Colorectal Polyp Cases and Features

Experienced and qualified proctologists performed electronic colonoscopy. Polyps would be normally removed when proctologists detected colorectal polyps (≥ 5 mm) during the electronic colonoscopy process. But, it would be not extirpated for poor bowel preparation, anticoagulant drug use, and patients with harsh refusal. Pathologic records of pathological sections were acquired by the Pathology Detection Department. The polyps were classified as proximal polyps (ascending colon, cecum, transverse colon, hepatic flexure, or splenic flexure), distal polyps (descending or sigmoid colon), and rectal polyps (rectum or rectosigmoid junction). The diameter of polyp < 10 mm was deemed as small polyps and diameter ≥ 10 mm was large polyps according to polyp size (20). Colorectal polyps were also classified as single polyp (number = 1) and multiple polyps (number > 1).

### Statistical Analysis

The energy density (g⋅2,000 kcal^–1^⋅d^–1^) of individual food intake was expressed via the well-known nutrient density method (21). Numbers with percentages (%) were used to express categorical variables and means ± standard errors were represented for continuous variables.

The ORs and 95% CIs of meat consumption were estimated by logistic regression models. Model 1 was adjusted for age and sex. Some important factors were additional adjustments in model 2, including BMI, alcohol consumption, physical activity, smoking status, annual household income, history of family colorectal cancer, and vitamin supplement use. Fruit consumption, fresh vegetables consumption, and total energy consumption were further adjusted for model 3 based on model 2. Healthy diet score and total energy consumption were additionally adjusted for model 4 on the basis of model 2. Each category median was utilized to analyze the linear trends.

We further assessed the relation of meat intake with different polyp prevalence according to anatomic location, size, and multiplicity. Sensitivity analyses were performed, including additional adjustment for other underlying confounders (calcium supplement use, aspirin medication use, and education levels) on the basis of model 4. The ORs and 95% CIs of meat consumption were computed after excluding participants with extreme BMI (< 18.5 or >40 kg/m^2^), with cancers, or with extreme energy (women: < 600 or >3,500 kcal/d and men: < 800 or >4,200 kcal/d), respectively. In order to assess whether a divergent association existed or not, subgroup analyses were also performed. The subgroup was classified according to sex (males or females), age (< 60 or ≥60 years), BMI (< 24 or ≥24 kg/m^2^), smoking status (non-smokers or smokers), physical activity (< median or ≥median), alcohol intake (non-drinkers or drinkers), healthy lifestyle score (< median or ≥median) and healthy diet score (< median or ≥median).

SAS 9.4 was used to perform statistical analyses. The analysis would be significant when the *P*-value of two-sided was < 0.05.

## Results

### Participants’ Characteristics

A total of 2,064 polyp cases were detected from March 2018 to December 2019. Participants who consumed more meat have a tendency to be younger, male, and alcohol drinkers. They were more educated, had more physical activity, and had higher annual household income. Participants with higher meat intake also consumed higher fruit and calories, less fresh vegetables, and dairy products (Table 1).

**TABLE 1:** Baseline characteristics of study participants according to quartiles of meat consumption (*n* = 6783)^a^.

	Quartiles of meat consumption (g⋅2,000 kcal^–1^⋅d^–1^)				
	
Characteristics	Q1^b^	Q2	Q3	Q4	*P*-value
*N*	1,695	1,696	1,696	1,696	
Age, *years*	61.2 ± 0.2	59.9 ± 0.2	59.6 ± 0.2	58.4 ± 0.2	**< 0.001**
Sex, *n* (%)					**0.002**
Male	808 (47.7)	878 (51.8)	913 (53.8)	899 (53.0)	
Female	887 (52.3)	818 (48.2)	783 (46.2)	797 (47.0)	
Family history of colorectal cancer, *n* (%)					0.142
No	1519 (89.6)	1510 (89.0)	1487 (87.7)	1524 (89.9)	
Yes	105 (6.2)	121 (7.1)	124 (7.3)	118 (7.0)	
Body mass index, *kg/m^2^*	23.3 ± 0.1	23.5 ± 0.1	23.5 ± 0.1	23.5 ± 0.1	0.107
<18.5, *n* (%)	940 (55.5)	909 (53.6)	919 (54.2)	936 (55.2)	
18.5–24.0, *n* (%)	89 (5.3)	63 (3.7)	76 (4.5)	61 (3.6)	
24.0–28, *n* (%)	542 (32.0)	596 (35.1)	562 (33.1)	571 (33.7)	
>28, *n* (%)	124 (7.3)	128 (7.6)	139 (8.2)	128 (7.6)	
Married, *n* (%)	1511 (89.1)	1573 (92.8)	1602 (94.5)	1610 (94.9)	**<0.001**
≥High school, *n* (%)	150 (8.9)	248 (14.6)	257 (15.2)	291 (17.2)	**<0.001**
Household income (yuan/yr), *n* (%)					**<0.001**
<30 thousand	735 (43.4)	515 (30.4)	487 (28.7)	454 (26.8)	
30–100 thousand	722 (42.6)	820 (48.4)	810 (47.8)	786 (46.3)	
>100 thousand	238 (14.0)	361 (21.3)	399 (23.5)	456 (26.9)	
Physical activity (MET-h/wk)^c^	175.5 ± 2.9	178.2 ± 2.9	180.1 ± 2.8	190.8 ± 2.9	**0.001**
Q1, *n* (%)	455 (26.8)	449 (26.5)	426 (25.1)	377 (22.2)	
Q2, *n* (%)	421 (24.8)	437 (25.8)	413 (24.4)	418 (24.7)	
Q3, *n* (%)	430 (25.4)	402 (23.7)	427 (25.2)	440 (25.9)	
Q4, *n* (%)	389 (23.0)	408 (24.1)	430 (25.4)	461 (27.2)	
Smoking status, *n* (%)					0.329
Never	1154 (68.1)	1103 (65.0)	1099 (64.8)	1095 (64.6)	
**Past smokers**					
<500/y	117 (6.9)	132 (7.8)	142 (8.4)	131 (7.7)	
>=500/y	88 (5.2)	105 (6.2)	81 (4.8)	112 (6.6)	
**Current smokers**					
<500/y	133 (7.9)	135 (8.0)	145 (8.6)	145 (8.6)	
>=500/y	203 (12.0)	221 (13.0)	229 (13.5)	213 (12.6)	
Alcohol drinker, *n* (%)					**<0.001**
Never	1017 (60.0)	900 (53.1)	890 (52.5)	922 (54.4)	
<=25 ml for men, <=15 ml for women	97 (5.7)	129 (7.6)	136 (8.0)	152 (9.0)	
>25 ml for men, >15 ml for women	581 (34.3)	667 (39.3)	670 (39.5)	622 (36.7)	
Vitamin supplement intake, *n* (%)					0.057
No	1681 (99.2)	1689 (99.6)	1684 (99.3)	1675 (98.8)	
Yes	14 (0.8)	7 (0.4)	12 (0.7)	21 (1.2)	
Calcium supplement intake, *n* (%)					0.904
No	1604 (94.6)	1610 (94.9)	1605 (94.6)	1613 (95.1)	
Yes	91 (5.4)	86 (5.1)	91 (5.4)	83 (4.9)	
Regular aspirin use, *n* (%)					0.796
No	1676 (98.9)	1681 (99.1)	1683 (99.2)	1677 (98.9)	
Yes	19 (1.1)	14 (0.8)	12 (0.7)	18 (1.1)	
History of hypertension, *n* (%)	491 (29.0)	462 (27.2)	425 (25.1)	368 (21.7)	**<0.001**
Cardiovascular disease, *n* (%)	18 (1.1)	24 (1.4)	11 (0.7)	9 (0.5)	**0.027**
Cancer, *n* (%)	20 (1.2)	19 (1.1)	18 (1.1)	24 (1.4)	0.792
Diabetes, *n* (%)	103 (6.1)	100 (5.9)	94 (5.5)	99 (5.8)	0.662
**Dietary intake**					
Total energy (kcal^–1^⋅d^–1^)	1916.6 ± 18.3	2034.5 ± 17.6	2054.8 ± 15.5	2057.2 ± 14.5	**<0.001**
Grain (g⋅2,000 kcal^–1^⋅d^–1^)	440.3 ± 2.1	414.2 ± 2.0	398.4 ± 1.6	357.7 ± 1.6	**<0.001**
Refined grain (g⋅2,000 kcal^–1^⋅d^–1^)	434.8 ± 2.1	409.3 ± 2.0	394.3 ± 1.6	352.4 ± 1.5	**<0.001**
Dairy products (g⋅2,000 kcal^–1^⋅d^–1^)	37.0 ± 2.1	34.6 ± 2.0	29.0 ± 1.6	29.8 ± 1.6	**0.005**
Total meat (g⋅2,000 kcal^–1^⋅d^–1^)	23.2 ± 0.3	57.1 ± 0.2	91.7 ± 0.3	164.0 ± 1.2	**<0.001**
Red meat (g⋅2,000 kcal^–1^⋅d^–1^)	13.9 ± 0.2	38.5 ± 0.4	68.5 ± 0.5	128.6 ± 1.3	**<0.001**
Unprocessed red meat (g⋅2,000 kcal^–1^⋅d^–1^)	13.6 ± 0.2	37.9 ± 0.4	67.9 ± 0.5	127.5 ± 1.3	**<0.001**
Processed red meat (g⋅2,000 kcal^–1^⋅d^–1^)	0.33 ± 0.02	0.51 ± 0.04	0.61 ± 0.04	1.12 ± 0.14	**<0.001**
Poultry (g⋅2,000 kcal^–1^⋅d^–1^)	2.5 ± 0.1	4.6 ± 0.1	5.9 ± 0.2	9.5 ± 0.4	**<0.001**
Seafood (g⋅2,000 kcal^–1^⋅d^–1^)	6.8 ± 0.2	14.0 ± 0.3	17.3 ± 0.4	26.4 ± 0.7	**<0.001**
Vegetables (g⋅2,000 kcal^–1^⋅d^–1^)	243.5 ± 2.7	231.8 ± 2.4	224.3 ± 2.3	220.4 ± 2.3	**<0.001**
Preserved vegetables (g⋅2,000 kcal^–1^⋅d^–1^)	4.2 ± 0.2	3.8 ± 0.2	3.4 ± 0.2	3.8 ± 0.3	0.161
Fruit (g⋅2,000 kcal^–1^⋅d^–1^)	94.4 ± 3.3	101.2 ± 2.3	105.1 ± 2.3	111.0 ± 2.3	**<0.001**

*^a^Data are percentages or means (standard errors) unless indicated otherwise.*

*^b^Q, quartile.*

*^c^MET-h/wk, metabolic equivalent task hours per week.*

### Meat and Meat Subtypes Consumption and Colorectal Polyp Prevalence

A multivariable-adjusted model was used to detect the associations of total meat, red meat, unprocessed and processed red meat, poultry, seafood consumption with the prevalence of colorectal polyps. Total meat intake was not related to polyp prevalence in the final model (*P*_for trend_ = 0.11). A positive association was found for poultry meat consumption in the model 1 [OR_Q4vs.Q1_ (95% CI): 1.28 (1.09–1.50); *P*_for trend_ = 0.002]. The relation was still significant after additional adjustment for demographic factors [OR_Q4vs.Q1_ (95% CI): 1.21 (1.02–1.42); *P*_for trend_ = 0.02] and was almost unchanged in model 3 [OR_Q4vs.Q1_ (95% CI): 1.20 (1.02–1.42); *P*_for trend_ = 0.03]. A significant relation of poultry meat consumption with polyp prevalence was still observed in model 4 [OR_Q4vs.Q1_ (95% CI): 1.20 (1.02–1.42); *P*_for trend_ = 0.03]. Higher processed red meat consumption was marginally related to a higher colorectal polyp prevalence [OR_Q4vs.Q1_ (95% CI): 1.15 (0.98–1.34); *P*_for trend_ = 0.06], whereas a similar association was not detected for total red meat (*P*_for trend_ = 0.81) and unprocessed red meat (*P*_for trend_ = 0.71). Seafood consumption was not related to colorectal polyp prevalence (Table 2).

**TABLE 2:** Multivariable-adjusted ORs (95% CIs) of meat consumption with the prevalence of colorectal polyps.

	Quartiles of meat consumption (g⋅2,000 kcal^–1^⋅d^–1^)				
	
Risk factors	Q1^a^	Q2	Q3	Q4	*P* for trend
**Total meat**					
Median (cut points, g⋅2,000 kcal^–1^⋅d^–1^)	24.2 (<40.7)	57.0 (40.7–73.6)	91.5 (73.6–111.7)	147.5 (≥111.7)	
Cases/*n*	515/1,695	497/1,696	536/1,696	516/1,696	
Model 1^b^	1 (ref)	0.96 (0.82–1.12)	1.08 (0.92–1.25)	1.08 (0.93–1.26)	0.16
Model 2^c^	1 (ref)	0.94 (0.80–1.10)	1.08 (0.92–1.26)	1.08 (0.93–1.27)	0.13
Model 3^d^	1 (ref)	0.94 (0.81–1.10)	1.08 (0.93–1.26)	1.09 (0.93–1.28)	0.12
Model 4^e^	1 (ref)	0.94 (0.80–1.09)	1.08 (0.92–1.26)	1.09 (0.93–1.28)	0.11
**Red meat**					
Median (cut points, g⋅2,000 kcal^–1^⋅d^–1^)	11.0 (<22.1)	35.7 (22.1–51.3)	68.8 (51.3–86.2)	116.8 (≥86.2)	
Cases/n	528/1,695	496/1,696	536/1,696	504/1,696	
Model 1	1 (ref)	0.93 (0.80–1.08)	1.00 (0.86–1.16)	0.97 (0.83–1.13)	0.93
Model 2	1 (ref)	0.92 (0.79–1.07)	0.99 (0.85–1.16)	1.00 (0.85–1.16)	0.80
Model 3	1 (ref)	0.91 (0.78–1.07)	0.98 (0.84–1.15)	1.00 (0.85–1.17)	0.82
Model 4	1 (ref)	0.91 (0.78–1.06)	0.99 (0.85–1.15)	1.00 (0.85–1.17)	0.81
**Unprocessed red meat**					
Median (cut points, g⋅2,000 kcal^–1^⋅d^–1^)	10.5 (<21.6)	35.0 (21.6–50.8)	68.0 (50.8–85.4)	115.8 (≥85.4)	
Cases/*n*	526/1695	495/1696	537/1696	506/1696	
Model 1	1 (ref)	0.94 (0.80–1.09)	1.00 (0.86–1.17)	0.98 (0.84–1.14)	0.97
Model 2	1 (ref)	0.92 (0.79–1.07)	1.00 (0.86–1.16)	1.01 (0.86–1.17)	0.70
Model 3	1 (ref)	0.92 (0.79–1.07)	0.99 (0.85–1.15)	1.01 (0.86–1.18)	0.72
Model 4	1 (ref)	0.92 (0.79–1.07)	0.99 (0.85–1.15)	1.01 (0.86–1.18)	0.71
**Processed red meat**					
Median (cut points, g⋅2,000 kcal^–1^⋅d^–1^)	0	0.3 (0.0–0.5)	0.7 (0.5–1.0)	1.5 (≥1.0)	
Cases/n	1090/3,623	319/1,053	333/1,054	322/1,053	
Model 1	1 (ref)	0.98 (0.84–1.14)	1.14 (0.98–1.33)	1.14 (0.97–1.33)	0.05
Model 2	1 (ref)	0.97 (0.83–1.13)	1.11 (0.95–1.30)	1.14 (0.97–1.33)	0.07
Model 3	1 (ref)	0.96 (0.82–1.12)	1.11 (0.95–1.30)	1.15 (0.98–1.35)	0.05
Model 4	1 (ref)	0.96 (0.82–1.12)	1.10 (0.94–1.29)	1.15 (0.98–1.34)	0.06
**Poultry**					
Median (cut points, g⋅2,000 kcal^–1^⋅d^–1^)	0 (<0.7)	1.7 (0.8–3.3)	4.0 (3.3–6.7)	14.3 (≥6.8)	
Cases/n	466/1,701	442/1,502	629/2,015	527/1,565	
Model 1	1 (ref)	1.11 (0.95–1.30)	1.18 (1.01–1.36)	1.28 (1.09–1.50)	**0.002**
Model 2	1 (ref)	1.09 (0.93–1.28)	1.14 (0.97–1.32)	1.21 (1.02–1.42)	**0.02**
Model 3	1 (ref)	1.09 (0.93–1.29)	1.14 (0.98–1.33)	1.20 (1.02–1.42)	**0.03**
Model 4	1 (ref)	1.09 (0.93–1.28)	1.13 (0.97–1.32)	1.20 (1.02–1.42)	**0.03**
**Seafood**					
Median (cut points, g⋅2,000 kcal^–1^⋅d^–1^)	1.6 (<3.3)	6.7 (3.5–10.7)	14.3 (11.4–20.8)	33.3 (≥21.4)	
Cases/*n*	568/1,950	418/1,436	494/1,585	584/1,812	
Model 1	1 (ref)	1.05 (0.89–1.22)	1.12 (0.96–1.30)	1.18 (1.02–1.37)	**0.02**
Model 2	1 (ref)	1.05 (0.90–1.23)	1.09 (0.93–1.27)	1.13 (0.97–1.31)	0.11
Model 3	1 (ref)	1.05 (0.90–1.23)	1.10 (0.94–1.29)	1.13 (0.97–1.32)	0.10
Model 4	1 (ref)	1.05 (0.89–1.23)	1.10 (0.94–1.28)	1.13 (0.97–1.31)	0.11

*^a^Q, quartile.*

*^b^Model 1 was adjusted for age and sex.*

*^c^Model 2 was further adjusted for BMI (<18.5, 18.5–24, 24–28, >28, in kg/m^2^), smoking (never, past smokers with <25 pack-years or ≥25 pack-years, current smokers with <25 pack-years or ≥25 pack-years), alcohol consumption (never, ≤25 ml for men and ≤15 ml for women, >25 ml for men and >15 ml for women), household annual income (yuan), physical activity (MET-h/wk), vitamin supplement use (yes or no), history of family colorectal cancer (yes or no) on the basis of model 1.*

*^d^Model 3 was further adjusted for total energy intake (quartile), intake of fruit and fresh vegetables (quartile) on the basis of model 2.*

*^e^Model 4 was further adjusted for total energy intake (quartile), healthy diet score (quartile) on the basis of model 2.*

In anatomic subsites of polyps, it was diagnosed 866 cases of proximal polyps, 900 cases of distal polyps, and 298 cases of rectum polyps (Supplementary Table 1). A positive relation of higher total meat intake with rectum polyp prevalence was found in the fully-adjusted model [OR_Q4vs.Q1_ (95% CI): 1.48 (1.03–2.11); *P*_for trend_ = 0.01]. Furthermore, a marginal associations with rectum polyp prevalence were detected for total red meat consumption [OR_Q4vs.Q1_ (95% CI):1.33 (0.94–1.90); *P*_for trend_ = 0.05] and unprocessed red meat [OR_Q4vs.Q1_ (95% CI): 1.30 (0.91–1.85); *P*_for trend_ = 0.04].

Additionally, higher processed red meat consumption had a relation with higher small polyp prevalence [OR_Q4vs.Q1_ (95% CI): 1.17 (0.99–1.38); *P*_for trend_ = 0.03]. For poultry, participants in the highest consumption also had a higher small polyp prevalence [OR_Q4vs.Q1_ (95% CI): 1.19 (1.00–1.41); *P*_for trend_ = 0.04] whereas no relation was found for large polyps [OR_Q4vs.Q1_ (95% CI): 1.28 (0.89–1.85); *P*_for trend_ = 0.25] (Supplementary Table 2). However, we did not observe significant relation of various meat consumption with large or small polyp prevalence except seafood consumption. A positive relation of seafood consumption with multiple polyps was observed [OR_Q4vs.Q1_ (95% CI): 1.70 (1.31–2.21); *P*_for trend_ < 0.001] (Supplementary Table 3).

### Sensitivity Analyses

The positive relation of poultry meat consumption with prevalent colorectal polyps was unchanged in sensitivity analyses, suggesting that our findings were reliable (Supplementary Table 4).

### Subgroup Analyses

The correlation of poultry consumption with prevalent colorectal polyps was similar in subgroups (*P* for interaction >0.05) and the relation of processed red meat consumption was similar in subgroups (*P* for interaction >0.05), which indicated baseline characteristics did seem to modify the documented associations. Noticeably, the association of poultry meat intake and polyps prevalence tended to be stronger among the elders aged ≥60 (*P* for interaction = 0.05) (Supplementary Table 5).

## Discussion

In the current analyses, participants with higher poultry meat consumption had a higher colorectal polyp prevalence, especially multiple polyp prevalence and small polyp prevalence. A marginal relation of processed red meat intake with polyp prevalence was found. Unprocessed red meat consumption and total meat consumption were related to a higher prevalence of rectum polyps.

Previous studies mainly focused on the role of processed meat intake or red meat intake and few studies detected the relation of various meat subtype consumption with colorectal polyp prevalence. The detrimental role of higher processed meat consumption and higher red meat consumption on colorectal adenoma risk was reported in several meta-analyses, and a dose-response relationship was found with the increase of red meat (100 g/day) or processed meat (50 g/day) (6, 7, 22). Additionally, another meta-analysis indicated that beef consumption was positively related to a higher polyp risk (23). In the current analysis, we found a marginal association for processed red meat consumption, which was generally in accordance with the above previous studies. A recently published case-control study, which utilized data from the Tennessee Colorectal Polyp Study, suggested that high intake of red [OR (95% CI): 2.38 (1.44–3.93)] and processed meat [OR (95% CI): 2.03 (1.30–3.17)] was strongly and positively related to sessile serrated lesion risk, and the association may partially be due to heterocyclic amine (HCA) intake (24). HCAs are produced during meat cooking at high temperatures, such as pan-frying, grilling, or barbecuing, and their reactive metabolites may cause DNA damage. Curing and smoking, as common methods of transforming fresh meat into processed meat, also induced carcinogenic chemicals, such as N-nitroso compounds (NOCs) (25). The carcinogenicity of NOCs has been observed in a variety of laboratory animals (26), and a line of previous studies suggested NOCs seemed to accelerate CRC development (27–29). It was uncovered that higher HCA intake could increase colorectal adenoma risk in two case-control studies (30, 31), which was supported by a meta-analysis (32). Furthermore, two HCAs intake, including 2-amino-3,8-dimethylimidazo[4,5-f]quinoxaline (MeIQx) and 2-amino-1-methyl-6-phenylimidazo[4,5-b]pyridine (PhIP), were reported to increase non-advanced adenoma risk [OR (95% CI): 1.18 (1.01–1.38) for MeIQx, OR (95% CI): 1.17 (1.10–1.35) for PhIP] (33).

Except for meat-derived mutagens and carcinogens, there were high proteins and important micronutrients (e.g., vitamins B, zinc, and iron) in red meat (34). Dietary iron consumption was inversely related to adenoma risk in a meta-analysis [relative risk (95% CI): 0.83 (0.71–0.98)] (35). Similar protective roles of total iron consumption from supplements and diet [OR (95% CI): 0.69 (0.56–0.86)] in prevalent distal colorectal adenoma were also reported in Prostate, Lung, Colorectal, and Ovarian Cancer Screening Trial (36). This may partly explain the reason why we did not find the relation of red meat consumption with prevalent polyps. Furthermore, the Chinese Dietary Guidelines (2016) recommended that 40–75 g/d meat consumption be appropriate. In the current population at a high risk of CRC, an average amount of red meat consumption (approximately 60 g/d) may not be enough to play a negative effect on developing colorectal polyps. Ethnic differences along with divergence in dietary patterns between the Western and Chinese populations should also be considered given that the current evidence was mainly derived from the Westerners. Despite no significant association with colorectal polyp prevalence, total meat and unprocessed red meat intake were both related to a higher rectum polyp risk. In the PLCO Cancer Screening Trial, well-done meat and grilled meat were all positively related to rectal adenoma risk (36). Moreover, compared to the colon, the rectum experienced a long time of transportation and fecal mass storage and was easily exposed to damages, including genotoxic and cytotoxic damages (37). Therefore, meat consumption may have a greater influence on the prevalence of rectum polyps.

No consensus has been reached regarding the relationship between fish and poultry consumption (referring to white meat) and colorectal polyp risk. According to the results of Nurses’ Health Study II, fish consumption was not related to colorectal adenoma prevalence [OR (95% CI): 0.96 (0.78–1.17)] whereas adolescence with higher poultry meat consumption had a lower total colorectal adenoma risk [OR (95% CI): 0.80 (0.64–0.99)] (38). Nonetheless, it was found that higher poultry meat consumption was positively related to adenoma risk (10, 30). Similarly, meta-analyses of observational studies reported different results from the findings in the current study. A meta-analysis included the evidence from 23 publications and found no significant association between colorectal adenomas prevalence and white meat [OR (95% CI): 0.96 (0.84–1.09)], poultry [OR (95% CI): 0.98 (0.80–1.18)], and fish [OR (95% CI): 0.98 (0.80–1.19)] intake (9). Another meta-analysis of prospective studies also suggested no relation of poultry consumption with colorectal adenoma or cancer risk (39), which was different from our main findings. It may be related to poultry with/without skin intake. Owing to the fact that the poultry with skin may be more grilled than poultry without skin, the higher level of HCA might be present in the poultry with skin (40). Moreover, it was suggested that HCA intake could increase the risk of the sessile serrated lesion [OR (95% CI): 2.48 (1.49–4.16)] in the Tennessee Colorectal Polyp Study (24). A case-control study found that poultry meat consumption had a positive association with the prevalence of colon cancer among Moroccan men [OR (95% CI): 1.27 (1.01–1.59)] (41). The difference in the range of meat intake, definition of poultry meat, and population composition may contribute to the null or negative findings. For instance, poultry included chicken and turkey in most studies, which was different from our definition of poultry as chicken, duck, and goose.

The overall dietary patterns may also be linked with the risk of polyps. For example, better adherence to the Mediterranean diet, which consumed less sugar-sweetened beverages and red meat and more fish [OR (95% CI): 0.66 (0.44–0.99)], were related to a lower odds of advanced polyps (42). A higher adherence to “prudent” pattern during high school, featuring a higher vegetables consumption, fruit consumption, and fish consumption, was related to a lower rectal adenoma risk [OR_Q5vs.Q1_ (95% CI): 0.45 (0.27–0.75); *P*_for trend_ = 0.005], but not colon adenomas (43). The above results may indirectly reflect the potential benefits of higher fish consumption under the high consumption of vegetables and fruit. To date, fish consumption has been found to decrease CRC risk in some studies (44, 45). Inversely, in studies conducted in Shanghai, China, eel, shrimp, shellfish (46), and fish (47) intake had a significant association with a higher CRC risk, which may support the positive relationship between seafood intake and higher odds of multiple polyps in the current Lanxi population. However, established findings related to CRC could not be extrapolated to adenoma or polyps, the precursors of CRC. To date, only one case-control study reported that the combination of fish and shellfish consumption was beneficial for a lower incidence of colon adenoma, proximal colon, and distal colon adenoma (48). Besides, Some confounding factors, including different ranges and types of fish consumed and racial differences, may influence the finding, which resulted in a null association with polyps. For instance, shellfish may be polluted by *Helicobacter pylori*, which was reported to be associated with a higher incidence of multiple colorectal adenomatous polyps [OR (95% CI): 2.38 (1.21–4.68); *P* < 0.05] (49). In addition, shellfish is abundant in cholesterol, while elevated serum triglyceride was correlated with the occurrence of colorectal polyps (50).

There are several advantages in the current study. The diagnosis of colorectal polyps included detailed histopathologic information, which allowed us to separately evaluate subsites, sizes, and multiplicity of polyps. Moreover, reverse causality was also minimized by assessing dietary factors and other key confounders such as a history of family colorectal cancer before an exam of personal electronic colonoscopy was taken. Most importantly, it was the first time to explore the relation of various subtypes of meat intake with colorectal polyps prevalence among the Chinese population.

Several limitations should be noted in the current analysis. First, colorectal adenomas and polyps were not analyzed individually, which would not provide more insight into the different progressing stages of CRC. Nonetheless, we further analyzed the association of subtypes of meat with small and large polyps, which could predict the possibility of a polyp developing into advanced neoplasia. Second, considering the observational study nature, residual or unmeasured confounding cannot be fully discarded. Third, the significant association may be diluted to the null relationship by unavoidable measurement errors. Fourth, although different seafood have different effects for human health, we did not separately collect dietary data of fish, shrimp, and shellfish and could not detect the effect of seafood subtypes intake on the occurrence of polyps. In addition, given that different ethnic groups have distinct dietary patterns and lifestyles, the generalization of our findings to other populations may be limited. Finally, given the observational study nature, a causal relationship cannot be achieved.

## Conclusion

Our current study investigated the relation of different subtypes of meat intake with the prevalence of polyps among the high-risk CRC Chinese population. The finding was that higher unprocessed red meat intake and total meat intake were related to a higher prevalent rectum polyp prevalence. Higher poultry consumption and processed red meat consumption were both related to an increased small polyp prevalence. Besides, a higher intake of poultry meat had a positive relationship with higher total colorectal polyp prevalence. Seafood consumption had a positive correlation with a higher multiple polyp prevalence. These findings provide emerging evidence supporting the current dietary guideline that recommends the low intake of poultry, processed and unprocessed red meat, and seafood for colorectal polyps prevention among the high-risk CRC Chinese population.

## Data Availability Statement

The raw data supporting the conclusions of this article will be made available by the authors, without undue reservation.

## Ethics Statement

The studies involving human participants were reviewed and approved by the Ethical Committee of Lanxi Red Cross Hospital. The patients/participants provided their written informed consent to participate in this study.

## Author Contributions

XC, ZY, PH, ST, and WZ: data collection. YL and FW: analysis and manuscript writing. YL, FW, XL, and PZ: data interpretation. JJ: study design. YZ, WZ, and JJ: critical revision of manuscript. All authors contributed to the article and approved the submitted version.

## Conflict of Interest

The authors declare that the research was conducted in the absence of any commercial or financial relationships that could be construed as a potential conflict of interest.

## Publisher’s Note

All claims expressed in this article are solely those of the authors and do not necessarily represent those of their affiliated organizations, or those of the publisher, the editors and the reviewers. Any product that may be evaluated in this article, or claim that may be made by its manufacturer, is not guaranteed or endorsed by the publisher.

## Abbreviations

BMI: body mass index
CRC: colorectal cancer
CI: confidence interval
HCA: heterocyclic amine
FFQ: food frequency questionnaire
LP3C: Lanxi Pre-colorectal Cancer Cohort
NOC: N-nitroso compounds
PHA: polycyclic aromatic hydrocarbons
OR: odds ratio
MET-h/wk: metabolic equivalent task hours per week.
