# Supplementary material for: Association of Meat Subtypes With Colorectal Polyp Prevalence: Finding From the Lanxi Pre-colorectal Cancer Cohort in China
Source: Front Nutr. 2022 Mar 18;9:833571. doi: 10.3389/fnut.2022.833571 (PMC8971953; doi:10.3389/fnut.2022.833571)
Supplement: Supplementary file 1 [file Data_Sheet_1.docx]

Supplementary Material

**Table of Contents**

**Supplement Table 1** Multivariable-adjusted ORs (95% CIs) of meat consumption with the prevalence of polyps according to polyp features

**Supplement Table 2** Multivariable-adjusted ORs (95% CIs) of meat consumption with the prevalence of polyps according to sizes

**Supplement Table 3** Multivariable-adjusted ORs (95% CIs) of meat consumption with the prevalence of polyps according to polyp numbers

**Supplement Table 4** Sensitivity analyses for multivariable-adjusted ORs (95% CIs) of meat consumption with the prevalence of polyps

**Supplement Table 5** Subgroup analyses for multivariable-adjusted ORs (95% CIs) of meat consumption with the prevalence of polyps

**Supplement Table 1.** Multivariable-adjusted ORs (95% CIs) of meat consumption with the prevalence of polyps according to polyp features ^a^

|  | **Anatomic Subsite** | | | | | | | |
| --- | --- | --- | --- | --- | --- | --- | --- | --- |
|  | **Proximal Colon** | |  | **Distal Colon** | |  | **Rectum** | |
| **Risk factors** | **Cases** | **OR (95% CI)** |  | **Cases** | **OR (95% CI)** |  | **Cases** | **OR (95% CI)** |
| **Total meat** |  |  |  |  |  |  |  |  |
| Q1 | 226 | 1 (ref) |  | 230 | 1 (ref) |  | 59 | 1 (ref) |
| Q2 | 221 | 0.95 (0.77-1.17) |  | 210 | 0.88 (0.71-1.08) |  | 66 | 1.11 (0.77-1.60) |
| Q3 | 211 | 0.96 (0.77-1.19) |  | 233 | 1.04 (0.84-1.28) |  | 92 | 1.64 (1.17-2.32) |
| Q4 | 208 | 1.02 (0.82-1.27) |  | 227 | 1.07 (0.86-1.32) |  | 81 | 1.48 (1.03-2.11) |
| *P* for trend |  | 0.87 |  |  | 0.30 |  |  | **0.01** |
| **Total red meat** |  |  |  |  |  |  |  |  |
| Q1 | 226 | 1 (ref) |  | 240 | 1 (ref) |  | 62 | 1 (ref) |
| Q2 | 232 | 1.00 (0.81-1.23) |  | 195 | 0.79 (0.64-0.98) |  | 69 | 1.09 (0.76-1.55) |
| Q3 | 210 | 0.89 (0.72-1.10) |  | 239 | 0.96 (0.79-1.18) |  | 87 | 1.41 (1.00-1.99) |
| Q4 | 198 | 0.91 (0.73-1.13) |  | 226 | 0.99 (0.80-1.22) |  | 80 | 1.33 (0.94-1.90) |
| *P* for trend |  | 0.25 |  |  | 0.68 |  |  | 0.05 |
| **Unprocessed red meat** |  |  |  |  |  |  |  |  |
| Q1 | 225 | 1 (ref) |  | 238 | 1 (ref) |  | 63 | 1 (ref) |
| Q2 | 231 | 1.00 (0.81-1.23) |  | 198 | 0.81 (0.66-1.00) |  | 66 | 1.02 (0.71-1.46) |
| Q3 | 210 | 0.89 (0.72-1.10) |  | 237 | 0.96 (0.78-1.17) |  | 90 | 1.43 (1.02-2.01) |
| Q4 | 200 | 0.93 (0.75-1.16) |  | 227 | 1.00 (0.81-1.24) |  | 79 | 1.30 (0.91-1.85) |
| *P* for trend |  | 0.33 |  |  | 0.65 |  |  | **0.04** |
| **Processed red meat** |  |  |  |  |  |  |  |  |
| Nonconsumers | 462 | 1 (ref) |  | 482 | 1 (ref) |  | 146 | 1 (ref) |
| T1 | 132 | 0.92 (0.73-1.14) |  | 132 | 0.89 (0.71-1.10) |  | 55 | 1.32 (0.95-1.83) |
| T2 | 149 | 1.16 (0.94-1.44) |  | 135 | 1.00 (0.81-1.24) |  | 49 | 1.24 (0.88-1.74) |
| T3 | 123 | 1.06 (0.84-1.32) |  | 151 | 1.19 (0.96-1.46) |  | 48 | 1.27 (0.90-1.79) |
| *P* for trend |  | 0.33 |  |  | 0.20 |  |  | 0.10 |
| **Poultry** |  |  |  |  |  |  |  |  |
| Q1 | 205 | 1 (ref) |  | 192 | 1 (ref) |  | 69 | 1 (ref) |
| Q2 | 163 | 0.91 (0.72-1.14) |  | 211 | 1.25 (1.00-1.56) |  | 68 | 1.14 (0.81-1.62) |
| Q3 | 278 | 1.13 (0.91-1.39) |  | 270 | 1.18 (0.95-1.45) |  | 81 | 1.03 (0.73-1.45) |
| Q4 | 220 | 1.11 (0.88-1.39) |  | 227 | 1.25 (1.00-1.57) |  | 80 | 1.32 (0.92-1.88) |
| *P* for trend |  | 0.17 |  |  | 0.10 |  |  | 0.21 |
| **Seafood** |  |  |  |  |  |  |  |  |
| Q1 | 248 | 1 (ref) |  | 236 | 1 (ref) |  | 84 | 1 (ref) |
| Q2 | 160 | 0.92 (0.74-1.15) |  | 203 | 1.21 (0.98-1.50) |  | 55 | 0.93 (0.65-1.33) |
| Q3 | 205 | 1.03 (0.83-1.28) |  | 219 | 1.17 (0.94-1.44) |  | 70 | 1.09 (0.77-1.52) |
| Q4 | 253 | 1.10 (0.89-1.36) |  | 242 | 1.12 (0.90-1.38) |  | 89 | 1.22 (0.88-1.70) |
| *P* for trend |  | 0.27 |  |  | 0.38 |  |  | 0.18 |

*^a^ Q, quartile; ORs, odds ratios; CIs, confidence intervals.*

**Supplement Table 2.** Multivariable-adjusted ORs (95% CIs) of meat consumption with the prevalence of polyps according to sizes ^a^

|  | **Size** | | | | |
| --- | --- | --- | --- | --- | --- |
|  | **<10 mm** | |  | **≥10 mm** | |
| **Risk factors** | **Cases** | **OR (95% CI)** |  | **Cases** | **OR (95% CI)** |
| **Total meat** |  |  |  |  |  |
| Q1 | 433 | 1 (ref) |  | 82 | 1 (ref) |
| Q2 | 434 | 0.97 (0.82-1.14) |  | 63 | 0.76 (0.54-1.08) |
| Q3 | 455 | 1.08 (0.92-1.27) |  | 81 | 1.07 (0.77-1.48) |
| Q4 | 443 | 1.10 (0.93-1.30) |  | 73 | 1.04 (0.74-1.47) |
| *P* for trend |  | 0.13 |  |  | 0.42 |
| **Total red meat** |  |  |  |  |  |
| Q1 | 446 | 1 (ref) |  | 82 | 1 (ref) |
| Q2 | 421 | 0.91 (0.78-1.08) |  | 75 | 0.90 (0.65-1.25) |
| Q3 | 464 | 1.01 (0.86-1.18) |  | 72 | 0.88 (0.63-1.23) |
| Q4 | 434 | 1.01 (0.86-1.19) |  | 70 | 0.92 (0.66-1.30) |
| *P* for trend |  | 0.66 |  |  | 0.62 |
| **Unprocessed red meat** |  |  |  |  |  |
| Q1 | 445 | 1 (ref) |  | 81 | 1 (ref) |
| Q2 | 420 | 0.92 (0.78-1.08) |  | 75 | 0.91 (0.66-1.27) |
| Q3 | 464 | 1.00 (0.85-1.18) |  | 73 | 0.89 (0.64-1.25) |
| Q4 | 436 | 1.02 (0.87-1.21) |  | 70 | 0.93 (0.66-1.31) |
| *P* for trend |  | 0.57 |  |  | 0.65 |
| **Processed red meat** |  |  |  |  |  |
| Nonconsumers | 923 | 1 (ref) |  | 167 | 1 (ref) |
| T1 | 271 | 0.96 (0.81-1.14) |  | 48 | 0.94 (0.67-1.33) |
| T2 | 291 | 1.14 (0.96-1.34) |  | 42 | 0.92 (0.65-1.32) |
| T3 | 280 | 1.17 (0.99-1.38) |  | 42 | 1.01 (0.71-1.45) |
| *P* for trend |  | **0.03** |  |  | 0.88 |
| **Poultry** |  |  |  |  |  |
| Q1 | 400 | 1 (ref) |  | 66 | 1 (ref) |
| Q2 | 373 | 1.07 (0.90-1.27) |  | 69 | 1.21 (0.85-1.72) |
| Q3 | 542 | 1.13 (0.96-1.33) |  | 87 | 1.14 (0.81-1.61) |
| Q4 | 450 | 1.19 (1.00-1.41) |  | 77 | 1.28 (0.89-1.85) |
| *P* for trend |  | **0.04** |  |  | 0.25 |
| **Seafood** |  |  |  |  |  |
| Q1 | 487 | 1 (ref) |  | 81 | 1 (ref) |
| Q2 | 349 | 1.02 (0.86-1.20) |  | 69 | 1.25 (0.89-1.75) |
| Q3 | 424 | 1.09 (0.93-1.28) |  | 70 | 1.14 (0.81-1.61) |
| Q4 | 505 | 1.13 (0.96-1.33) |  | 79 | 1.10 (0.78-1.56) |
| *P* for trend |  | 0.11 |  |  | 0.67 |

*^a^ Q, quartile; ORs, odds ratios; CIs, confidence intervals.*

**Supplement Table 3.** Multivariable-adjusted ORs (95% CIs) of meat consumption with the prevalence of polyps according to polyp numbers ^a^

| **Risk factors** | **Multiplicity** | | | | |
| --- | --- | --- | --- | --- | --- |
|  | **Single** | |  | **Multiple** | |
|  | **Cases** | **OR (95% CI)** |  | **Cases** | **OR (95% CI)** |
| **Total meat** |  |  |  |  |  |
| Q1 | 373 | 1 (ref) |  | 142 | 1 (ref) |
| Q2 | 373 | 0.97 (0.82-1.15) |  | 124 | 0.84 (0.64-1.09) |
| Q3 | 422 | 1.17 (0.99-1.38) |  | 114 | 0.82 (0.62-1.07) |
| Q4 | 373 | 1.08 (0.91-1.28) |  | 143 | 1.14 (0.87-1.48) |
| *P* for trend |  | 0.13 |  |  | 0.41 |
| **Total red meat** |  |  |  |  |  |
| Q1 | 381 | 1 (ref) |  | 147 | 1 (ref) |
| Q2 | 362 | 0.92 (0.78-1.09) |  | 134 | 0.89 (0.69-1.15) |
| Q3 | 430 | 1.10 (0.93-1.31) |  | 106 | 0.67 (0.51-0.88) |
| Q4 | 368 | 0.99 (0.84-1.18) |  | 136 | 1.01 (0.77-1.31) |
| *P* for trend |  | 0.53 |  |  | 0.50 |
| **Unprocessed red meat** |  |  |  |  |  |
| Q1 | 380 | 1 (ref) |  | 146 | 1 (ref) |
| Q2 | 363 | 0.93 (0.78-1.10) |  | 132 | 0.88 (0.68-1.14) |
| Q3 | 428 | 1.10 (0.93-1.30) |  | 109 | 0.69 (0.52-0.91) |
| Q4 | 370 | 1.01 (0.85-1.20) |  | 136 | 1.02 (0.79-1.33) |
| *P* for trend |  | 0.49 |  |  | 0.61 |
| **Processed red meat** |  |  |  |  |  |
| Nonconsumers | 807 | 1 (ref) |  | 283 | 1 (ref) |
| T1 | 242 | 1.00 (0.84-1.18) |  | 77 | 0.85 (0.64-1.12) |
| T2 | 255 | 1.14 (0.97-1.36) |  | 78 | 0.98 (0.74-1.29) |
| T3 | 237 | 1.14 (0.95-1.35) |  | 85 | 1.19 (0.91-1.55) |
| *P* for trend |  | 0.07 |  |  | 0.37 |
| **Poultry** |  |  |  |  |  |
| Q1 | 352 | 1 (ref) |  | 114 | 1 (ref) |
| Q2 | 351 | 1.15 (0.97-1.37) |  | 91 | 0.89 (0.66-1.19) |
| Q3 | 459 | 1.12 (0.94-1.32) |  | 170 | 1.18 (0.91-1.54) |
| Q4 | 379 | 1.17 (0.97-1.40) |  | 148 | 1.29 (0.98-1.72) |
| *P* for trend |  | 0.14 |  |  | **0.02** |
| **Seafood** |  |  |  |  |  |
| Q1 | 447 | 1 (ref) |  | 121 | 1 (ref) |
| Q2 | 310 | 0.99 (0.83-1.18) |  | 108 | 1.29 (0.97-1.71) |
| Q3 | 393 | 1.11 (0.94-1.31) |  | 101 | 1.05 (0.79-1.40) |
| Q4 | 391 | 0.98 (0.82-1.16) |  | 193 | 1.70 (1.31-2.21) |
| *P* for trend |  | 0.90 |  |  | **<0.001** |

*^a^ Q, quartile; ORs, odds ratios; CIs, confidence intervals.*

**Supplement Table 4.** Sensitivity analyses for multivariable-adjusted ORs (95% CIs) of meat consumption with the prevalence of polyps ^a^

| **Risk factors** | **Quartiles of meat consumption (g · 2000 kcal^-1^· d^-1^)** | | | | ***P* for trend** |
| --- | --- | --- | --- | --- | --- |
|  | **Q1** | **Q2** | **Q3** | **Q4** |  |
| **Excluding persons with history of cancer** |  |  |  |  |  |
| Cases/n | 2040/6702 |  |  |  |  |
| Total meat | 1 (ref) | 0.93 (0.79-1.09) | 1.07 (0.92-1.25) | 1.09 (0.93-1.28) | 0.12 |
| Red meat | 1 (ref) | 0.91 (0.78-1.06) | 0.98 (0.84-1.15) | 0.99 (0.84-1.16) | 0.89 |
| Unprocessed red meat | 1 (ref) | 0.91 (0.78-1.07) | 0.98 (0.84-1.15) | 1.00 (0.85-1.17) | 0.79 |
| Processed red meat | 1 (ref) | 0.96 (0.82-1.13) | 1.09 (0.93-1.28) | 1.15 (0.98-1.35) | 0.07 |
| Poultry | 1 (ref) | 1.07 (0.91-1.26) | 1.12 (0.96-1.31) | 1.20 (1.01-1.42) | **0.03** |
| Seafood | 1 (ref) | 1.06 (0.90-1.24) | 1.11 (0.95-1.29) | 1.15 (0.98-1.34) | 0.07 |
| **Excluding persons with extreme BMI** |  |  |  |  |  |
| Cases/n | 1993/6492 |  |  |  |  |
| Total meat | 1 (ref) | 0.94 (0.80-1.10) | 1.11 (0.95-1.30) | 1.09 (0.93-1.29) | 0.09 |
| Red meat | 1 (ref) | 0.91 (0.78-1.06) | 0.99 (0.85-1.16) | 1.00 (0.85-1.17) | 0.77 |
| Unprocessed red meat | 1 (ref) | 0.91 (0.78-1.07) | 1.00 (0.85-1.16) | 1.01 (0.86-1.18) | 0.69 |
| Processed red meat | 1 (ref) | 0.95 (0.81-1.12) | 1.08 (0.92-1.27) | 1.14 (0.97-1.34) | 0.10 |
| Poultry | 1 (ref) | 1.13 (0.96-1.34) | 1.18 (1.01-1.38) | 1.23 (1.04-1.46) | **0.01** |
| Seafood | 1 (ref) | 1.06 (0.90-1.24) | 1.08 (0.92-1.26) | 1.12 (0.96-1.31) | 0.15 |
| **Excluding persons with extreme energy** |  |  |  |  |  |
| Cases/n | 2018/6682 |  |  |  |  |
| Total meat | 1 (ref) | 0.92 (0.78-1.07) | 1.09 (0.93-1.27) | 1.10 (0.94-1.29) | 0.08 |
| Red meat | 1 (ref) | 0.90 (0.77-1.06) | 1.00 (0.86-1.17) | 1.00 (0.85-1.17) | 0.69 |
| Unprocessed red meat | 1 (ref) | 0.91 (0.78-1.06) | 1.00 (0.86-1.17) | 1.01 (0.86-1.19) | 0.61 |
| Processed red meat | 1 (ref) | 0.96 (0.82-1.13) | 1.11 (0.95-1.30) | 1.13 (0.97-1.33) | 0.07 |
| Poultry | 1 (ref) | 1.08 (0.92-1.28) | 1.13 (0.97-1.32) | 1.20 (1.01-1.42) | **0.03** |
| Seafood | 1 (ref) | 1.05 (0.90-1.24) | 1.11 (0.94-1.29) | 1.12 (0.96-1.31) | 0.13 |
| **Further adjusted for calcium supplement** |  |  |  |  |  |
| Cases/n | 2064/6783 |  |  |  |  |
| Total meat | 1 (ref) | 0.94 (0.80-1.09) | 1.08 (0.92-1.26) | 1.09 (0.93-1.28) | 0.11 |
| Red meat | 1 (ref) | 0.91 (0.78-1.06) | 0.99 (0.85-1.15) | 1.00 (0.85-1.17) | 0.81 |
| Unprocessed red meat | 1 (ref) | 0.92 (0.79-1.07) | 0.99 (0.85-1.15) | 1.01 (0.86-1.18) | 0.71 |
| Processed red meat | 1 (ref) | 0.96 (0.82-1.12) | 1.10 (0.94-1.29) | 1.14 (0.98-1.34) | 0.07 |
| Poultry | 1 (ref) | 1.09 (0.93-1.28) | 1.13 (0.97-1.32) | 1.20 (1.02-1.42) | **0.03** |
| Seafood | 1 (ref) | 1.05 (0.89-1.23) | 1.10 (0.94-1.28) | 1.12 (0.96-1.31) | 0.12 |
| **Further adjusted for aspirin use** |  |  |  |  |  |
| Cases/n | 2064/6783 |  |  |  |  |
| Total meat | 1 (ref) | 0.94 (0.80-1.09) | 1.08 (0.92-1.26) | 1.09 (0.93-1.28) | 0.12 |
| Red meat | 1 (ref) | 0.91 (0.78-1.06) | 0.99 (0.85-1.15) | 0.99 (0.85-1.16) | 0.83 |
| Unprocessed red meat | 1 (ref) | 0.92 (0.79-1.07) | 0.99 (0.85-1.15) | 1.00 (0.86-1.18) | 0.74 |
| Processed red meat | 1 (ref) | 0.96 (0.82-1.12) | 1.10 (0.94-1.28) | 1.15 (0.98-1.35) | 0.06 |
| Poultry | 1 (ref) | 1.09 (0.92-1.28) | 1.13 (0.97-1.32) | 1.20 (1.02-1.42) | **0.03** |
| Seafood | 1 (ref) | 1.05 (0.90-1.23) | 1.09 (0.94-1.28) | 1.13 (0.97-1.32) | 0.11 |
| **Further adjusted for education level** |  |  |  |  |  |
| Cases/n | 2064/6783 |  |  |  |  |
| Total meat | 1 (ref) | 0.94 (0.80-1.09) | 1.07 (0.92-1.25) | 1.09 (0.93-1.28) | 0.11 |
| Red meat | 1 (ref) | 0.91 (0.78-1.07) | 0.99 (0.84-1.15) | 1.00 (0.85-1.17) | 0.79 |
| Unprocessed red meat | 1 (ref) | 0.92 (0.79-1.07) | 0.99 (0.84-1.15) | 1.01 (0.86-1.18) | 0.70 |
| Processed red meat | 1 (ref) | 0.96 (0.82-1.12) | 1.10 (0.94-1.29) | 1.15 (0.98-1.34) | 0.07 |
| Poultry | 1 (ref) | 1.08 (0.92-1.28) | 1.13 (0.97-1.32) | 1.19 (1.01-1.41) | **0.04** |
| Seafood | 1 (ref) | 1.05 (0.89-1.23) | 1.10 (0.94-1.28) | 1.12 (0.96-1.31) | 0.12 |

*^a^ Q, quartile; ORs, odds ratios; CIs, confidence intervals.*

**Supplement Table 5** Subgroup analyses for multivariable-adjusted ORs (95% CIs) of meat consumption with the prevalence of polyps ^a^

| **Subgroups** | **Cases/*n*** | **Quartiles of meat consumption (g · 2000 kcal^-1^· d^-1^)** | | | | ***P-*Trend** | ***P* for interaction** |
| --- | --- | --- | --- | --- | --- | --- | --- |
|  |  | **Q1** | **Q2** | **Q3** | **Q4** |  |  |
| **Poultry** |  |  |  |  |  |  |  |
| **Age** |  |  |  |  |  |  | 0.05 |
| <60 y | 786/3344 | 1 (ref) | 0.90 (0.69-1.17) | 0.85 (0.67-1.09) | 1.02 (0.79-1.31) | 0.91 |  |
| ≥60 y | 1278/3439 | 1 (ref) | 1.22 (0.99-1.50) | 1.38 (1.13-1.68) | 1.32 (1.06-1.65) | 0.004 |  |
| **Sex** |  |  |  |  |  |  | 0.55 |
| Men | 1391/3498 | 1 (ref) | 1.08 (0.86-1.35) | 1.10 (0.90-1.35) | 1.16 (0.94-1.44) | 0.18 |  |
| Women | 673/3285 | 1 (ref) | 1.09 (0.86-1.38) | 1.19 (0.94-1.50) | 1.25 (0.95-1.65) | 0.07 |  |
| **BMI** |  |  |  |  |  |  | 0.86 |
| <24 | 1118/3993 | 1 (ref) | 1.13 (0.92-1.40) | 1.25 (1.02-1.53) | 1.14 (0.91-1.43) | 0.13 |  |
| ≥24 | 946/2790 | 1 (ref) | 1.01 (0.78-1.31) | 0.99 (0.77-1.25) | 1.25 (0.97-1.60) | 0.11 |  |
| **Physical activity** |  |  |  |  |  |  | 0.48 |
| <Median | 1082/3381 | 1 (ref) | 1.18 (0.95-1.47) | 1.16 (0.93-1.44) | 1.11 (0.88-1.41) | 0.41 |  |
| ≥Median | 982/3402 | 1 (ref) | 0.94 (0.74-1.20) | 1.11 (0.89-1.38) | 1.27 (1.01-1.61) | 0.02 |  |
| **Smoking** |  |  |  |  |  |  | 0.69 |
| Nonsmoker | 1082/4451 | 1 (ref) | 1.00 (0.82-1.23) | 1.13 (0.93-1.38) | 1.13 (0.91-1.40) | 0.17 |  |
| Former/current smoker | 982/2332 | 1 (ref) | 1.26 (0.96-1.65) | 1.17 (0.91-1.51) | 1.33 (1.02-1.73) | 0.08 |  |
| **Alcohol consumption** |  |  |  |  |  |  | 0.75 |
| Nondrinker | 1010/3729 | 1 (ref) | 0.99 (0.80-1.22) | 1.09 (0.89-1.34) | 1.19 (0.94-1.49) | 0.12 |  |
| Drinker | 1054/3054 | 1 (ref) | 1.23 (0.95-1.60) | 1.21 (0.95-1.53) | 1.26 (0.98-1.61) | 0.12 |  |
| **Healthy diet score** |  |  |  |  |  |  | 0.98 |
| <Median | 958/3003 | 1 (ref) | 1.13 (0.89-1.44) | 1.13 (0.90-1.42) | 1.23 (0.96-1.58) | 0.13 |  |
| ≥Median | 1106/3780 | 1 (ref) | 1.06 (0.85-1.32) | 1.14 (0.93-1.40) | 1.17 (0.94-1.47) | 0.13 |  |
| **Healthy lifestyle score** |  |  |  |  |  |  | 0.84 |
| <Median | 1543/4768 | 1 (ref) | 1.08 (0.89-1.31) | 1.07 (0.89-1.29) | 1.17 (0.96-1.42) | 0.14 |  |
| ≥Median | 521/2015 | 1 (ref) | 1.14 (0.83-1.55) | 1.33 (1.00-1.78) | 1.32 (0.95-1.84) | 0.05 |  |
|  |  |  |  |  |  |  |  |
| **Processed red meat** |  |  |  |  |  |  |  |
| **Age** |  |  |  |  |  |  | 0.93 |
| <60 y | 786/3344 | 1 (ref) | 1.14 (0.90-1.45) | 1.34 (1.06-1.69) | 1.10 (0.87-1.40) | 0.11 |  |
| ≥60 y | 1278/3439 | 1 (ref) | 0.83 (0.67-1.03) | 0.94 (0.76-1.17) | 1.22 (0.98-1.51) | 0.29 |  |
| **Sex** |  |  |  |  |  |  | 0.88 |
| Men | 1391/3498 | 1 (ref) | 0.98 (0.81-1.19) | 1.16 (0.95-1.42) | 1.12 (0.91-1.38) | 0.14 |  |
| Women | 673/3285 | 1 (ref) | 0.93 (0.70-1.24) | 1.01 (0.78-1.30) | 1.19 (0.93-1.52) | 0.24 |  |
| **BMI** |  |  |  |  |  |  | 0.61 |
| <24 | 1118/3993 | 1 (ref) | 0.96 (0.77-1.19) | 1.05 (0.85-1.30) | 1.10 (0.89-1.37) | 0.37 |  |
| ≥24 | 946/2790 | 1 (ref) | 0.96 (0.76-1.22) | 1.15 (0.91-1.45) | 1.20 (0.94-1.52) | 0.09 |  |
| **Physical activity** |  |  |  |  |  |  | 0.29 |
| <Median | 1082/3381 | 1 (ref) | 1.08 (0.87-1.36) | 1.17 (0.94-1.46) | 1.23 (0.98-1.53) | 0.04 |  |
| ≥Median | 982/3402 | 1 (ref) | 0.83 (0.66-1.05) | 1.03 (0.82-1.28) | 1.06 (0.84-1.33) | 0.66 |  |
| **Smoking** |  |  |  |  |  |  | 0.53 |
| Nonsmoker | 1082/4451 | 1 (ref) | 0.96 (0.77-1.20) | 1.19 (0.97-1.45) | 1.18 (0.96-1.44) | 0.05 |  |
| Former/current smoker | 982/2332 | 1 (ref) | 0.95 (0.75-1.20) | 1.00 (0.78-1.28) | 1.12 (0.87-1.44) | 0.49 |  |
| **Alcohol consumption** |  |  |  |  |  |  | 0.84 |
| Nondrinker | 1010/3729 | 1 (ref) | 0.91 (0.72-1.15) | 1.07 (0.85-1.34) | 1.18 (0.95-1.46) | 0.16 |  |
| Drinker | 1054/3054 | 1 (ref) | 0.99 (0.79-1.23) | 1.13 (0.90-1.40) | 1.11 (0.88-1.39) | 0.26 |  |
| **Healthy diet score** |  |  |  |  |  |  | 0.17 |
| <Median | 958/3003 | 1 (ref) | 0.96 (0.76-1.21) | 1.04 (0.82-1.32) | 1.08 (0.85-1.39) | 0.47 |  |
| ≥Median | 1106/3780 | 1 (ref) | 0.99 (0.77-1.28) | 1.23 (0.97-1.57) | 1.26 (0.99-1.60) | 0.03 |  |
| **Healthy lifestyle score** |  |  |  |  |  |  | 0.46 |
| <Median | 1543/4768 | 1 (ref) | 0.95 (0.79-1.14) | 1.04 (0.87-1.25) | 1.14 (0.95-1.37) | 0.19 |  |
| ≥Median | 521/2015 | 1 (ref) | 0.98 (0.68-1.40) | 1.36 (0.97-1.91) | 1.19 (0.85-1.67) | 0.12 |  |

*^a^ Q, quartile; ORs, odds ratios; CIs, confidence intervals.*
